# Clinical Characteristics of Inpatients with Anaphylaxis in China

**DOI:** 10.1155/2015/429534

**Published:** 2015-05-04

**Authors:** Rui Tang, Han-Yi Xu, Ju Cao, Shi Chen, Jin-Lu Sun, Hong Hu, Hai-Chao Li, Ying Diao, Zhi Li

**Affiliations:** ^1^Department of Allergy, Peking Union Medical College Hospital, Chinese Academy of Medical Sciences & Peking Union Medical College, Beijing 100730, China; ^2^Department of Respiratory Medicine, The General Hospital of Chinese People's Liberation Army, Beijing 100853, China; ^3^Department of Respiratory Medicine, Peking University First Hospital, No. 8 Xishiku Street, Xicheng District, Beijing 100034, China; ^4^Department of Endocrinology, Peking Union Medical College Hospital, Chinese Academy of Medical Sciences & Peking Union Medical College, Beijing 100730, China

## Abstract

*Objective.* To analyze the clinical characteristics of inpatients with anaphylaxis and the factors that influenced those characteristics.* Methods.* Using the patient records from 1990 to 2013 from three highly ranked Chinese hospitals, we retrospectively analyzed the characteristics of 108 inpatients with anaphylaxis (not anaphylaxis admitted).* Results.* The mean patient age was 42 ± 20 years old and male-to-female ratio was 1 : 1.3. The number of patients with anaphylaxis increased gradually, and cases diagnosed after 2005 accounted for 68.5% of the 108 total cases. The most common trigger was medications. The most common clinical manifestations included cutaneous, nervous, respiratory, circulatory, and digestive signs and symptoms. Male patients were more likely to experience loss of consciousness. Multisystem involvement was more likely to develop in patients with low BP, whereas it was uncommon in those with anaphylaxis induced by antibiotics or anesthetics. Epinephrine was used as the first-line treatment for 56 cases.* Conclusions.* Inpatient with anaphylaxis was more common in female patients and the number increased gradually during the study period. The most common trigger was medications. Patients with low BP were prone to having multisystem involvement, whereas the cases of anaphylaxis induced by antibiotics and anesthetics were less likely to involve multiple organ systems.

## 1. Introduction

Anaphylaxis is a severe and life-threatening allergic reaction that involves multiple organ systems or the whole body and has an incidence rate of 0.05%–2% [1]. Acute episodes are usually attributed to type I hypersensitivity (immediate hypersensitivity) mediated by IgE. The condition can involve the skin, mucosa, respiratory tract, cardiovascular system, and the digestive tract [2]. Anaphylaxis attacks can cause blood pressure to drop within minutes to a few hours and can be lethal if emergency treatment is not provided in a timely manner. In this retrospective study, we review the clinical manifestations and influencing factors of inpatients with anaphylaxis in these 3 well recognized hospitals in Beijing, aiming to help improve early recognition, diagnosis, and treatment of anaphylaxis in clinical practice.

## 2. Materials and Methods

### 2.1. Patients

A total of 108 patients were diagnosed with anaphylaxis from 1990 to 2013 at the Peking Union Medical College Hospital (42 cases) and Peking University First Hospital (22 cases) and from 1993 to 2013 at the General Hospital of the Chinese People's Liberation Army (44 cases).

### 2.2. Methods

In this retrospective study, all existing records were analyzed retrospectively and anonymously. We selected patients by discharge diagnosis codes; those admitted for anaphylaxis were excluded. We summarized the general patient condition, clinical manifestations during attacks, and factors that influenced the manifestations, including the relationships between gender, age, etiology, and underlying diseases with the laboratory and clinical manifestations of anaphylaxis and among clinical manifestations, the relationship between blood pressure and multisystem involvement. The study had been approved by ethics committee.

Based on the criteria of the American Academy of Allergy, Asthma and Immunology (AAAAI), the American College of Allergy, Asthma and Immunology (ACAAI), and the Joint Council of Allergy, Asthma and Immunology (JCAAI) [3], anaphylaxis was diagnosed if the patient presented one of the following 3 clinical scenarios: (a) acute onset (minutes to hours) with involvement of the skin and mucosa, as well as airway obstruction, reduced blood pressure, or symptoms of hypovolemia; (b) at least 2 of the following occurring within minutes or a few hours after contact with or exposure to an allergen: cutaneous and mucosal involvement, airway obstruction, a drop in blood pressure, symptoms of hypovolemia, or gastrointestinal symptoms; (c) reduced blood pressure after exposure to an allergen (systolic pressure <70 mmHg in 1–12-month-old infants, <70 mmHg + 2 × age in 1–10-year-old children, <90 mmHg in 11–17-year-old adolescents, or reduced by over 30% compared to the baseline blood pressure).

The clinical presentations of anaphylaxis mainly involve the skin, respiratory tract, digestive tract, circulatory system, and nervous system. Patients were divided into 2 groups, one with at least 3 systems involved and the other with only 1 or 2 systems involved. The blood pressure and laboratory test results obtained during the episodes and during baseline conditions (times when the patients were not experiencing anaphylaxis attacks) were compared. Routine laboratory tests were conducted using an automatic biochemical analyzer and an automatic hematology analyzer.

### 2.3. Statistical Analysis

The data were all tested for normal distributions. Normally distributed data were expressed as means ± standard deviation, and paired-sample *t*-tests were applied to compare blood pressure and heart rate during episodes and during baseline conditions. Data that did not fit a normal pattern of distribution were expressed as medians (i.e., P25 and P75). Among independent samples, normally distributed data were analyzed using a *t*-test and abnormally distributed data were analyzed using a rank-sum test. Constituent ratios were compared using the chi-square test. *P* values <0.05 represented statistically significant differences.

## 3. Results

### 3.1. General Patient Information

The 108 patients in this study were 42 ± 20 years old (range: 3–76 years), including 46 males and 62 females (male-to-female ratio: 1 : 1.3).

Among the anaphylaxis cases, 42 were inpatients from Peking Union Medical College Hospital, 44 were from the General Hospital of the Chinese People's Liberation Army, and 22 were from Peking University First Hospital. Anaphylaxis cases accounted for 0.005% of the 827,791 and 0.003% of the 786,732 inpatients were treated at the Peking Union Medical College Hospital and Peking University First Hospital, respectively, from 1990 to 2013, and 0.006% of the 796970 at the General Hospital of the Chinese People's Liberation Army from 1993 to 2013.

The cases were 7, 15, 11, 30, and 45 during 1990–1994, 1995–1999, 2000–2004, 2005–2009, and 2010–2013, so the incidence was 0.005%, 0.006%, 0.003%, 0.005%, and 0.004%, respectively.

We evaluated the blood pressure and heart rate during anaphylaxis attacks. Baseline blood pressure was recorded for 105 cases. Blood pressure during attacks was recorded for 102 cases, of which 20 were unobtainable (systolic and diastolic pressures were both recorded as 0 mmHg). The systolic pressure decreased from 117.4 ± 13.8 mmHg during baseline conditions to 54.3 ± 31.9 mmHg during episodes (*P* < 0.01) and the diastolic pressure decreased from 71.6 ± 13.6 mmHg during baseline conditions to 33.9 ± 21.4 mmHg during episodes (*P* < 0.01). Baseline heart rate records were available for all of the patients (81 ± 13 bpm), and the heart rates during episodes were recorded for 78 patients (107 ± 38 bpm) and were found to be significantly increased (*P* < 0.01).

The cause of anaphylaxis was unclear for 4 cases (3.7%). In 97 cases (89.8%), anaphylaxis was presumably triggered by medications, specifically antibiotics in 32 cases (29.6%)—including penicillin, ciprofloxacin, cefoperazone sulbactam, cefmetazole, cravit, or cefaclor; contrast media in 18 cases (16.7%); chemotherapy drugs in 12 cases (11.1%)—including L-asparaginase, sulfur hexafluoride, and cisplatin; anesthetics in 8 cases (7.4%); nutritional support in 7 cases (6.5%); blood products in 6 cases (5.6%); antipyretic analgesics in 4 cases (3.7%); Chinese patent medicines in 3 cases (2.8%); a glucocorticoid in 1 case (0.9%); a vaccine in 1 case (0.9%); and other drugs in 5 cases (4.6%). In the remaining 7 cases, the trigger of anaphylaxis was smell in 1 case (0.9%) and food in 6 cases (5.6%), such as steamed bun of Chinese chive, protein powder, peanut, snapping turtle, wheat, and lactic acid milk biscuit. The symptoms of anaphylaxis developed immediately or within 6 hours after allergen exposure.

Twenty-four patients experienced intraoperative anaphylaxis, including 11 during gynecologic surgery, 3 during otorhinolaryngologic surgery, 2 during pancreatic surgery, 2 during urologic surgery, 2 during cardiac surgery, and 1 each during thyroid, ophthalmic, breast, and gallbladder surgeries. Severe allergic reactions to chemotherapy drugs, antibiotics, anesthetics drugs, and nutritional support were detected in 4 cases, including 2 cases caused by blood transfusion, 1 caused by hemostatic drugs, and 1 caused by glucocorticoids. A history of food and/or drug allergies was positively reported in 28 patients and was not reported for the other 80 patients. Eight patients had a history of acute urticaria, bronchial asthma, or allergic rhinitis.

### 3.2. Clinical Manifestations of Anaphylaxis and Influencing Factors

Clinical manifestations were presented as follows in the 108 patients: 78 developed skin signs (72.2%), mainly rashes, itching, wheals, redness, and swelling; 59 had nervous system signs (54.6%), primarily loss of consciousness and syncope; 57 had respiratory signs (52.8%), mainly shortness of breath, dyspnea, and wheezing in all areas of the lungs; 45 had circulatory signs (41.7%), including pale face, palpitation, excessive sweating, lip cyanosis, and dizziness; and 41 had gastrointestinal signs (38.0%), including nausea, vomiting, abdominal pain, diarrhea, and fecal incontinence. Rashes, dyspnea, and loss of consciousness were the most common signs of anaphylaxis (Table 1).

The relationship between gender and the most common clinical manifestations was as follows: the female-to-male ratio of patients who developed rashes was higher than that of patients with no rash (42 : 25 versus 20 : 21, *P* < 0.05), was significantly lower in patients with dyspnea compared with those who did not have dyspnea (23 : 27 versus 39 : 19, *P* < 0.05), and was also significantly lower in patients presenting loss of consciousness compared with those who did not present this sign (18 : 23 versus 44 : 23, *P* < 0.05).

The relationship between blood pressure and the most common clinical manifestations was as follows: blood pressure was unobtainable in 20 cases, of which 11 (55.0%) had rashes and 11 (55.0%) had dyspnea, neither of which were significantly different from the percentages of the patients with obtainable blood pressure (62.9% and 43.8%); of the patients with unobtainable blood pressure, 55.0% experienced a loss of consciousness, which was significantly higher than that in patients with obtainable blood pressure (33.7%, *P* < 0.05).

For multisystem involvement, the clinical manifestations of anaphylaxis mainly involved the skin, respiratory system, circulatory system, digestive system, and nervous system. In the 108 patients, 53 presented signs in at least 3 systems and 55 presented signs in 1 or 2 systems; comparisons between these groups are shown in Table 2.

### 3.3. Characteristics of Laboratory Test Results during Anaphylaxis

We used paired-sample *t*-tests to analyze the following characteristics during both baseline conditions and anaphylaxis episodes: white blood cell and neutrophil tests in 53 patients, percentage and absolute count of eosinophils in 35 patients, blood glucose levels in 38 patients, alanine transaminase and total bilirubin levels in 32 patients, creatinine and blood urea nitrogen levels in 40 patients, serum potassium levels in 50 patients, serum sodium levels in 49 patients, serum chloride levels in 47 patients, and serum calcium levels in 36 patients.

As shown in Table 3, both the white blood cell count and the neutrophil percentage increased significantly during episodes of anaphylaxis (both *P* = 0.000), so did the decrease of eosinophil percentage (*P* = 0.000). A significant increase was detected in the blood glucose levels (*P* = 0.000), but not in the alanine transaminase, total bilirubin, creatinine, or blood urea nitrogen levels. Serum potassium and calcium levels both decreased during anaphylaxis (*P* = 0.001, 0.040), while no significant difference was observed for serum sodium or chloride levels.

### 3.4. Treatment and Prognoses

Patients were mainly treated with epinephrine and glucocorticoids, which alleviated the anaphylaxis condition in minutes or a few days. Epinephrine, glucocorticoids, and antihistamine were received among 56, 94, and 25 patients, respectively. Improvement or recovery was observed in all cases except for 6 deaths (5.6%). In these 6 patients, who were 43 ± 20 years old, 5 had visited one of the 3 hospitals in this study during the previous 10 years, 2 developed anaphylaxis during an operation, and 1 had a history of bronchial asthma. All episodes were triggered by medication, including 2 by antibiotics, 2 by chemotherapy drugs, 1 by a nutritional supplement, and 1 by a glucocorticoid; 6 patients experienced loss of consciousness and 3 patients had multisystem involvement.

## 4. Discussion

Anaphylaxis is a severe, rapidly progressing, systemic allergic reaction, usually with multisystem involvement, and is life-threatening if not immediately treated. The patients in this study all had acute onset after contact with or exposure to an allergen immediately or within 6 hours and presented with cutaneous, respiratory, circulatory, gastrointestinal, or nervous symptoms and signs. Patients experienced significant drops in blood pressure compared to baseline conditions, leading to a definitive diagnosis of anaphylaxis according to the criteria of the AAAAI, ACAAI, and JCAAI (revised in 2010) [3].

According to Lieberman [1], the incidence rate of anaphylaxis is 0.05%–2%. However, the incidence rate of anaphylaxis among inpatients in the Peking Union Medical College Hospital, the General Hospital of the Chinese People's Liberation Army, and Peking University First Hospital in this study was 0.005%, 0.006%, and 0.003%, respectively. The reason of stable incidence was due to the increasing of inpatient number. The low incidence maybe has relationship with excluding out-of-hospital anaphylaxis. A retrospective study from Bangkok, Thailand, reported only 6% anaphylaxis developed during hospitalization, with estimated incidence 0.0011% (5/448211) [4]. As Sheikh and Alves [5] and Gupta et al. [6] reported, the incidence rate of anaphylaxis in 2003-2004 was 7 times higher than in 1990-1991. Although the incidence was low and stable, the number of patients with anaphylaxis increases gradually in this 24-year, 3-centre study, with instances of anaphylaxis occurring after 2005 accounting for 68.5% of all cases. The possible mechanisms for the increasing number of inpatients maybe are as follows: the clinicians pay more attention to anaphylaxis, guidelines increase clinicians' understanding of anaphylaxis, and the types of drugs and testing methods are more and more. This time trend reminds us of the urgency of preventing anaphylaxis, along with the need for technical progress in treating episodes.

Women are affected more often than men [7]. In the present study, most of the anaphylaxis patients were female (62/108). The causes maybe are that women have more chance to experience operation, such as induced abortion, Caesarea, or uterine adnexectomy. The systolic and diastolic pressures during anaphylaxis attacks significantly decreased compared to the baseline conditions (*P* < 0.01), which might have resulted from increased vascular permeability or vascular smooth muscle relaxation induced by histamine release [8]. Heart rates were significantly higher during attacks than during baseline conditions (*P* < 0.01), which might be caused by the decrease in the effective blood volume or by accelerated diastolic depolarization of the sinoatrial node induced by histamine-H1 receptor interactions [9].

Identifying the cause of anaphylaxis is crucial for the targeted prevention of episodes, and medications were by far the most common cause [10, 11]. Except for 4 cases in which the cause was unclear, most of the 108 cases of anaphylaxis in this study were triggered by medications, with some episodes occurring during operations. Considering the rapid development and wide use of new drugs, especially antibiotics, and the common practice of prophylactic antimicrobial treatment for patients undergoing an operation, the most common cause of drug-induced anaphylaxis is therefore antibiotics. With the rising incidence of cardiovascular and cerebrovascular diseases and tumors, contrast media, chemotherapy drugs, nutritional support solutions, blood products, and antipyretic analgesics have also become common triggers of anaphylaxis. The mechanisms underlying drug-induced anaphylaxis include both allergic and nonallergic reactions, with the former being IgE-mediated or non-IgE-mediated and the latter including directional release of mediators by mast cells and basophils, activation of the contact system, disturbances of arachidonic acid metabolism, and recruitment of complement factors, coagulation factors, and fibrinolytic factors. Among these triggers, antibiotics and anesthetics could induce IgE-mediated allergic reactions, contrast media could induce contact system activation and complement-mediated reactions, both anesthetics and contrast media could activate mast cells, and aspirin and nonsteroidal anti-inflammatory drugs could disrupt arachidonic acid metabolism. Given the mechanisms of drug-induced anaphylaxis, tryptase measurements, blood and/or urine histamine tests, and basophil degranulation tests have thus been the primary diagnostic methods for anaphylaxis [12–14]. Among nondrug triggers, food is the most common cause of anaphylaxis and is also the cause of most out-of-hospital anaphylaxis cases [15]. Although the rate of incidence is lower, food-induced anaphylaxis is often not treated as promptly as drug-induced anaphylaxis in inpatients and thus may be more dangerous.

The most common clinical manifestation of anaphylaxis is on the skin, including urticaria and angioedema, which are observed in 85%–90% cases of anaphylaxis; the most common respiratory sign is edema of the upper respiratory tract, observed in 50%–60% cases; 25%–30% cases present gastrointestinal signs, including nausea, vomiting, diarrhea, and cramp-like abdominal pain; 30%–35% cases have dizziness, syncope, and a blood pressure drop; and a few patients present headache, substernal chest pain, or epileptic seizures [9]. Similar frequencies of clinical manifestations were observed in this study, with skin signs being the most common (72.2%), followed by neurological (54.6%), respiratory (52.8%), circulatory (41.7%), and digestive tract (38.0%) signs.

Respiratory signs and loss of consciousness were more often observed in male patients. Because the patients who experience a blood pressure drop are more likely to develop circulatory and neurological signs, lower diastolic and systolic pressures are correlated with a higher possibility of multisystem involvement. Intraoperative anaphylaxis is seldom associated with multisystem involvement, most likely because under general anesthesia the occurrence of anaphylaxis is usually detected based on a blood pressure drop and reduced oxygen saturation, as shown on monitors, or by a wheal-like rash over the torso or limbs observed after removing sterile drapes. Anaphylaxis in these cases is not based on patient-reported chest distress, shortness of breath, dyspnea, abdominal pain, nausea, vomiting, dizziness, or syncope, in contrast to patients with full consciousness.

Increased white blood cell counts, neutrophil percentages, and blood glucose levels were observed in this study in anaphylaxis patients, which is presumably the result of stress and elevated blood catecholamine levels. A reduction in serum potassium levels during anaphylaxis attacks was also noticed, likely because high catecholamine levels would induce potassium transport into the cells and produce a transient decrease in serum potassium [16]. The reduced serum calcium levels observed in this study have not been reported in previous literature. It has been suggested that the reduction in serum calcium levels in severe anaphylaxis patients might be related to suppressed thyroid function, ineffective activated vitamin D, calcium chelators, and hypomagnesemia or be caused by calcium redistribution induced by inflammatory factors [17].

The first choice treatment for anaphylaxis is the timely administration of epinephrine [1, 18, 19]. Only 56 of 108 patients received epinephrine treatment in the study. The cause maybe is that clinicians in the other departments lack understanding about manifestation and treatment of anaphylaxis. So this is the importance of the study. With the progress of medical technology and an improved understanding of anaphylaxis, the mortality rate associated with this condition has been decreasing. Patients with a history of allergic disease or who present with a loss of consciousness and multisystem involvement have a high risk of death. Therefore, it is important to immediately and correctly recognize, diagnose, and treat anaphylaxis based on knowledge of the clinical manifestations and an understanding of the factors that influence anaphylaxis.

This is, as far as we know, the first inpatient anaphylaxis study among three highly ranked hospitals in China. In this study, we had a few limitations, which were lack of tryptase and sIgE detection. Anaphylaxis is a clinical diagnosis that builds on the clinical criteria [20]. Retrospectively the diagnosis may be supported if serum tryptase is elevated within a few hours after the reaction when compared with the patient's baseline levels; however, tryptase levels did not all increase obviously in anaphylaxis [21, 22]. As for sIgE detection, it can be tested for a few antibiotics in this study and allergy knowledge should be updated for doctors in other specialties. Out-of-hospital anaphylaxis maybe more often happened than inpatient anaphylaxis in our three hospitals, so we will take these patients into account in the further study.

## 5. Conclusion

In conclusion, the anaphylaxis inpatients studied in the 3 hospitals were more common in female patients. The number of inpatients with anaphylaxis exhibited a rising trend over the study period. During anaphylaxis attacks, the patients presented significantly decreased blood pressure and increased heart rates. The most common causes were drugs, especially antibiotics. The most common clinical manifestations were skin signs; respiratory, neurological, circulatory, and gastrointestinal signs were also common. Male patients were more prone to develop respiratory signs and lose consciousness. The patients presenting a drop in blood pressure during anaphylaxis attacks were more likely to have multisystem involvement, whereas those patients with anaphylaxis induced by antibiotics or anesthetics were less likely to have multisystem involvement.

## Figures and Tables

**Table 1:** Clinical manifestations of anaphylaxis in the inpatients studied [*n* (%)].

Clinical manifestation	Number (% or percent)
Rash	67 (62.0)
Dyspnea	50 (46.3)
Loss of consciousness	41 (38.0)
Nausea/vomiting	30 (27.8)
Pale face/lip cyanosis	27 (25.0)
Excessive sweating	23 (21.3)
Palpitation	20 (18.5)
Abdominal pain	14 (13.0)
Facial swelling	13 (12.0)
Lung rale	11 (10.2)
Dizziness	11 (10.2)
Convulsion	9 (8.3)
Impalpable pulse	7 (6.5)
Blurred vision	5 (4.6)
Diarrhea/fecal incontinence	4 (3.7)
Coughing	2 (1.9)
Skin peeling	1 (0.9)

**Table 2:** Clinical characteristics of anaphylaxis patients with multisystem involvement.

	Multisystem involvement (*n* = 53)	1-2 system involvement (*n* = 55)	*P* value
Age (yr, *x* ± *s*)	40 ± 21	44 ± 19	NS
Systolic BP during anaphylaxis attacks [mm Hg, median (LQ, UQ)]	33 (0, 40)	68 (50, 80)	<0.05
Diastolic BP during anaphylaxis attacks [mm Hg, median (LQ, UQ)]	26 (0, 35)	45 (30, 48)	<0.05
Female : male ratio	28 : 25	34 : 21	NS
Surgical cases	8	16	<0.05
History of allergy	13	15	NS
Antibiotic- or anesthetic-induced anaphylaxis	16	24	<0.05
Food-induced anaphylaxis	5	1	NS

BP: blood pressure; NS: not significant.

**Table 3:** Routine laboratory test results during baseline conditions and during anaphylaxis attacks.

Test results	*N*	Baseline condition	Anaphylaxis attack	*P* value
WBC count (×10^9^/L, $\overset{-}{x}\pm s$)	53	8.2 ± 4.2	13.9 ± 7.6	0.000
Neutrophil percentage (%, $\overset{-}{x}\pm s$)	53	64.3 ± 17.4	78.5 ± 20.0	0.000
Eosinophil percentage [%, median (LQ, UQ)]	35	0.60 (0.01, 2.15)	0.01 (0.00, 0.10)	0.000
Blood glucose (mmol/L, $\overset{-}{x}\pm s$)	38	6.1 ± 2.7	11.8 ± 4.5	0.000
Alanine transaminase [U/L, median (LQ, UQ)]	32	8.4 (6.3, 12.4)	10.1 (6.9, 14.0)	NS
Total bilirubin (mmol/L, $\overset{-}{x}\pm s$)	32	9.6 ± 4.3	10.0 ± 4.4	NS
Blood creatinine (mmol/L, $\overset{-}{x}\pm s$)	40	79.3 ± 39.7	81.2 ± 49.4	NS
Blood urea nitrogen (mmol/L, $\overset{-}{x}\pm s$)	40	6.1 ± 7.9	6.4 ± 8.0	NS
Serum potassium (mmol/L, $\overset{-}{x}\pm s$)	50	4.0 ± 0.4	3.6 ± 0.7	0.001
Serum sodium (mmol/L, $\overset{-}{x}\pm s$)	49	137.8 ± 6.6	138.4 ± 5.4	NS
Serum chloride (mmol/L, $\overset{-}{x}\pm s$)	47	104.2 ± 5.2	104.5 ± 6.3	NS
Serum calcium (mmol/L, $\overset{-}{x}\pm s$)	36	2.27 ± 0.41	2.12 ± 0.48	0.040

WBC: white blood cell.
